# Temporal trends and epidemiological impact of metabolic risk factors on stroke burden in Chinese individuals aged 65 and older, 1992–2021

**DOI:** 10.3389/fneur.2025.1607823

**Published:** 2025-07-29

**Authors:** Haiying Wang, Xiaofeng Wang, Jia Wang, Lin Geng, Shaohua Hou, Pu Li, Qiang Wang, Yali Cui, Shengli Sun

**Affiliations:** ^1^Department of Science and Education, Shenmu Hospital, The Affiliated Shenmu Hospital of Northwest University, Shenmu, China; ^2^Department of Emergency, Ruijin Hospital, Shanghai Jiao Tong University School of Medicine, Shanghai, China; ^3^Department of Neurology, Shenmu Hospital, The Affiliated Shenmu Hospital of Northwest University, Shenmu, China; ^4^College of Life Sciences, Northwest University, Xi’an, China; ^5^Department of Orthopedics, Shenmu Hospital, The Affiliated Shenmu Hospital of Northwest University, Shenmu, China

**Keywords:** stroke, metabolic risk factors, age-standardized deaths rate, autoregressive integrated moving average (ARIMA) models, population aging

## Abstract

**Background:**

Stroke remains a principal cause of mortality and disability globally, with approximately 75 percent of strokes affecting individuals aged 65 and older. High fasting plasma glucose (HFPG), high LDL cholesterol (LDL-C), and high systolic blood pressure (SBP) are significant metabolic risk factors contributing to the increasing stroke burden. Despite this, the specific impact of these factors on stroke distribution within China, the world’s most populous aging nation, has not been extensively explored. This study investigates the influence of HFPG, LDL-C, and SBP on the stroke burden among China’s elderly population from 1992 to 2021.

**Methods:**

Utilizing data from the Global Burden of Disease (GBD) 2021 database, this study assesses the trends in stroke burden attributable to metabolic risks among Chinese individuals aged 65 years and older from 1992 to 2021. Joinpoint regression analysis was employed to identify changes in age-standardized deaths rate (ASDR), while age-period-cohort (APC) analysis helped delineate the roles of age, period, and cohort effects on stroke mortality. Future projections up to 2031 were estimated using autoregressive integrated moving average (ARIMA) models.

**Results:**

The study period saw a decline in age-standardized rates (ASRs) of stroke-related mortality and disability-adjusted life years (DALYs) across all metabolic risk factors, indicating progress in healthcare and public health efforts. Despite these improvements, the absolute numbers of deaths and DALYs continued to rise, propelled by an aging population and increasing prevalence of metabolic disorders. High SBP [1,241,130 (95% UI: 785,358–1,711,112)] was identified as the most significant contributor to stroke-related mortality among the elderly in China, followed by HFPG [243,757 (95% UI: 175,821–324,600)] and LDL-C [249,106 (95% UI: 70,548–449,517)]. Projections suggest that while ASDR may decrease, the absolute number of stroke-related deaths linked to these metabolic risks will continue to increase through 2031.

**Conclusion:**

Developing effective population-specific strategies targeting high SBP, HFPG, and LDL-C is crucial for substantially reducing the stroke burden among China’s elderly. Given the significant population-attributable fractions of these risk factors, it is vital for China to rigorously evaluate its disease burden and implement targeted prevention and control strategies to mitigate future impacts.

## Introduction

Stroke stands as a primary cause of mortality and disability globally, exerting a particularly heavy toll on the elderly population (1, 2). As demographic trends shift toward an aging global populace, the incidence of strokes linked to modifiable metabolic risk factors—namely high fasting plasma glucose (HFPG), high low-density lipoprotein cholesterol (LDL-C), and high systolic blood pressure (SBP)—is on the rise (3–6). These factors significantly contribute to the global disease burden, positioning them at the forefront of public health intervention strategies (7).

In China, which hosts the world’s largest aging demographic, significant changes in stroke epidemiology have occurred over the last 30 years (8). These changes are largely attributable to rapid urbanization, evolving lifestyle patterns, and healthcare advancements, collectively influencing stroke incidence and outcomes (9, 10). Despite reductions in the age-standardized deaths rate (ASDR) and age-standardized disability-adjusted life years (DALYs) due to stroke, the absolute burden continues to escalate, propelled by demographic shifts and a rising tide of metabolic disorders (11). For example, the increasing prevalence of diabetes, hypertension, and dyslipidemia during the past decade. High SBP has been consistently pinpointed as the predominant factor driving this burden, followed by HFPG and high LDL-C (3, 5). Tu et al. (10) projected the overall age-standardized prevalence, incidence, and mortality rates for stroke of the Chinese population aged 40 years or older in 2020 were 2.6%, 505.2 per 100,000 person-years, and 343.4 per 100,000 person-years, respectively (10, 12). The prevalence was significantly higher among individuals over 60 years of age compared to those under 60 (12). The number of first ever strokes per year (3.4 million) was more than the number per year in the US (0.61 million) and the European Union (1.12 million) (12–14).

Addressing the dynamic landscape of stroke burden linked to metabolic risks is crucial for developing tailored prevention and management strategies (15). Among the six major metabolic risk factors identified in the Global Burden of Disease (GBD) 2021 database, high SBP, HFPG and high LDL-C have been consistently recognized as the most influential contributors to stroke mortality and DALYs in both global and regional assessments (4, 8, 16). Therefore, this study aims to thoroughly examine the trends, disparities, and future projections of stroke burden attributable to these three metabolic risk factors among individuals aged 65 years and older in China from 1992 to 2021. Utilizing data from the GBD 2021 database, our analysis offers vital insights into the impact of metabolic risks on stroke outcomes, underscoring the need for focused public health initiatives and informed policy-making. A 10-year projection through 2031 was performed using the autoregressive integrated moving average (ARIMA) model to support medium-term health planning while balancing trend sensitivity and forecast reliability (17).

## Methods

### Data source

The stroke data analyzed in this study are obtained from the GBD 2021 utilizing the GBD Results Tool, conducted by the Institute for Health Metrics and Evaluation (IHME), which provides a comprehensive scientific assessment of published, publicly available, and contributory incidence, prevalence, and mortality data for 369 diseases, injuries, and impairments, as well as 88 risk factors across 21 GBD regions and 204 countries and territories (18). The primary objective of GBD is to generate consistent, transparent, and systematic estimates of disease burden across regions and over time, to inform health policy and resource allocation globally. Data inputs to the GBD include vital registration systems, household surveys, disease registries, health service utilization data, scientific literature, and other administrative sources. The burden estimation process employs sophisticated statistical modeling tools (18). In this study, we extracted data on stroke burden attributable to three key metabolic risk factors—HFPG, high LDL-C, and high SBP—among individuals aged ≥ 65 years in China, covering the period from 1992 to 2021. To summarize the age distribution of the burden of stroke among individuals aged ≥ 65 years, patients were divided into 7 groups: 65–69 years, 70–74 years, 75–79 years, 80–84 years, 85–89 years, 90–94 years, ≥95 years (19). Stroke was defined corresponding to ICD-10 codes I60-I63 and ICD-9 codes 430–434, encompassing both ischemic and hemorrhagic subtypes. The data were accessed through the GBD Results Tool, which allows users to customize queries by age, sex, location, year, cause, and risk factor. Detailed descriptions of these data sources and their validation processes have been systematically reviewed and can be accessed via the Global Health Data Exchange web tool.^1^

### Temporal trends

Joinpoint regression analysis, a linear statistical model used to analyze temporal trends in disease burden, was applied to detect significant shifts in ASDR trends from 1992 to 2021 (20). The annual percentage change (APC) and its 95% confidence interval (CI) were calculated for each metabolic risk, providing insight into the pace and direction of change over time (21). The APC is calculated as APC = [(y_x + 1_^_^ y_x_)/y_x_] * 100% = (e^β1 _^ 1) * 100%, evaluating the trend of independent intervals of piecewise functions. Trends were examined separately for males and females to capture sex-specific differences in stroke burden.

### Age-period-cohort analysis

The age-period-cohort (APC) framework was employed to disentangle the effects of age, time period, and birth cohort on stroke mortality (22, 23). The Wald *χ*^2^ test was performed to assess the statistical significance of trends in annual percentage change (24). Longitudinal age curves describe age- and cohort-specific rates, adjusted for period bias, and are generally regarded as superior to cross-sectional age curves in assessing age effects. Relative risk (RR) for a cohort (or period) represents the cohort’s RR (or period’s RR), adjusted for age and nonlinear cohort (or period) effects, in comparison with the reference cohort (or period). When the RR value exceeds 1, it indicates that the factor elevates the risk of stroke mortality. Conversely, when the RR value is <1, it suggests that the factor reduces the risk of stroke mortality.

In a typical APC model, the age and period intervals must be of equal duration. Consequently, seven distinct 5-year age groups (65–69, 70–74, 75–79, 80–84, 85–89, 90–94, and ≥95 years) were adopted for further analysis. The period spanning 1992 to 2021 was divided into six 5-year intervals (1992–1996, 1997–2001, 2002–2006, 2007–2011, 2012–2016, and 2017–2021). As a result, 12 consecutive 5-year birth cohort groups were identified (1897–1901, 1902–1906, 1907–1911, 1912–1916, 1917–1921, 1922–1926, 1927–1931, 1932–1936, 1937–1941, 1942–1946, 1947–1951, 1952–1956).

### Prediction analysis

To estimate the burden of stroke attribute to high SBP, HFPG and high LDL-C among old people in the next 10 years, the deaths number, and ASDR of pancreatitis among people aged ≥ 65 years were predicted utilizing the ARIMA models (24). Firstly, download the demographic data of stroke in the elderly from 1992 to 2021 demographic data from the GBD 2021 database, and access the predicted demographic data in the database. Next, utilize the R package to forecast the future trend burden of pancreatitis among the elderly.

### Statistical analysis

The Joinpoint Regression Program (version 4.9.1.0) was utilized to evaluate trends in stroke from 1990 to 2021. ARIMA analysis and plot generation were conducted using R (version 4.3.2) with the packages “forecast,” “magrittr,” “reshape2,” and “ggplot2.” A *p*-value of <0.05 was considered statistically significant.

## Results

### Stroke burden attributable to metabolic risks in 1992 and 2021

#### HFPG

From 1992 to 2021, total deaths increased from 91,341 (95% UI: 65,379–121,031) to 243,757 (95% UI: 175,821–324,600), while the ASDR declined from 179.32 (95% UI: 128.4–237.93) to 149.67 (95% UI: 107.71–198.99). DALYs increased in absolute terms, from 1,642,131 (95% UI: 1,176,575–2,159,899) to 4,140,456 (95% UI: 3,021,513–5,494,620), though the age-standardized DALYs decreased from 2,761.47 (95% UI: 1,983.45–3,635.85) to 2,342.83 (95% UI: 1,706.53-3,105.59). YLDs and YLLs also rose, with YLDs increasing from 106,608 (95% UI: 68,573–155,269) to 470,202 (95% UI: 305,977–671,973) and YLLs from 1,535,523 (95% UI: 1,095,380–2,034,026) to 3,670,254 (95% UI: 2,649,192–4,905,234) (Table 1).

**Table 1:** Burden of stroke attribute to metabolic risks among people aged ≥65 years in China in 1992 and 2021.

Risk factor	Measure	1992		2021	
		All-ages cases	Age-standardized rates per 100,000 people	All-ages cases	Age-standardized rates per 100,000 people
		*n* (95% UI)	*n* (95% UI)	*n* (95% UI)	*n* (95% UI)
HFPG	Deaths	91,341 (65,379, 121,031)	179.32 (128.4, 237.93)	243,757 (175,821, 324,600)	149.67 (107.71, 198.99)
	DALYs	1,642,131 (1,176,575, 2,159,899)	2761.47 (1983.45, 3635.85)	4,140,456 (3,021,513, 5,494,620)	2342.83 (1706.53, 3105.59)
	YLDs	106,608 (68,573, 155,269)	164.85 (105.57, 240.21)	470,202 (305,977, 671,973)	253.63 (164.94, 362.89)
	YLLs	1,535,523 (1,095,380, 2,034,026)	2596.62 (1855.85, 3442.95)	3,670,254 (2,649,192, 4,905,234)	2089.2 (1506.34, 2786.95)
High LDL-C	Deaths	85,216 (25,406, 156,988)	164.86 (47.3, 307.38)	249,106 (70,548, 449,517)	152.8 (42.58, 277.49)
	DALYs	1,624,398 (498,729, 2,945,255)	2665.94 (790.87, 4896.59)	4,458,305 (1,302,211, 7,956,535)	2498.91 (717.73, 4490.78)
	YLDs	156,155 (45,705, 294,099)	233.43 (66.23, 446.15)	655,692 (195,626, 1,234,466)	347.85 (102.1,659.07)
	YLLs	1,468,243 (449,256, 2,679,961)	2432.51 (719.79, 4489.96)	3,802,613 (1,105,665, 6,787,733)	2151.06 (615.26, 3866.38)
High SBP	Deaths	571,352 (336,373, 803,433)	1074.5 (614.84, 1524.65)	1,241,130 (785,358, 1,711,112)	748.9 (470.87, 1037.84)
	DALYs	10,240,455 (6,047,200, 14,374,034)	16675.76 (9690.63, 23514.11)	20,896,598 (13,302,307, 28,606,846)	11656.91 (7396.87, 16010.43)
	YLDs	411,561 (226,140, 636,990)	625.93 (340.83, 971.73)	1,734,721 (1,045,510, 2,581,155)	928.16 (555.85, 1381.28)
	YLLs	9,828,894 (5,799,940, 13,832,799)	16049.83 (9317.89, 22688.14)	19,161,877 (12,137,640, 26,323,061)	10728.75 (6776.1, 14788.8)

DALYs, Disability-Adjusted Life Years; YLDs, Years Lived with Disability; YLLs, Years of Life Lost; UI, uncertainty interval; HFPG, High fasting plasma glucose; High LDL-C, high LDL cholesterol; High SBP, High systolic blood pressure.

#### High LDL-C

Deaths attributed to high LDL-C increased from 85,216 (95% UI: 25,406–156,988) to 249,106 (95% UI: 70,548–449,517), with a corresponding decrease in the ASDR (164.86 to 152.8 per 100,000). DALYs rose in total from 1,624,398 (95% UI: 498,729–2,945,255) to 4,458,305 (95% UI: 1,302,211–7,956,535), but the age-standardized rate (ASR) decreased from 2,665.94 (95% UI: 790.87–4,896.59) to 2,498.91 (95% UI: 717.73–4,490.78). Similarly, YLDs increased from 156,155 (95% UI: 45,705–294,099) to 655,692 (95% UI: 195,626–1,234,466), and YLLs from 1,468,243 (95% UI: 449,256–2,679,961) to 3,802,613 (95% UI: 1,105,665–6,787,733) (Table 1).

#### High SBP

For high SBP, deaths increased from 571,352 (95% UI: 336,373–803,433) to 1,241,130 (95% UI: 785,358–1,711,112), while the ASDR decreased from 1,074.5 (95% UI: 614.84–1,524.65) to 748.9 (95% UI: 470.87–1,037.84). DALYs increased from 10,240,455 (95% UI: 6,047,200–14,374,034) to 20,896,598 (95% UI: 13,302,307–28,606,846), with the ASR decreasing from 16,675.76 (95% UI: 9,690.63–23,514.11) to 11,656.91 (95% UI: 7,396.87–16,010.43). YLDs and YLLs also saw substantial increases, with YLDs rising from 411,561 (95% UI: 226,140–636,990) to 1,734,721 (95% UI: 1,045,510–2,581,155), and YLLs from 9,828,894 (95% UI: 5,799,940–13,832,799) to 19,161,877 (95% UI: 12,137,640–26,323,061) (Table 1).

### Age and sex differences in stroke burden attributable to metabolic risks in 2021

**Figure 1:**
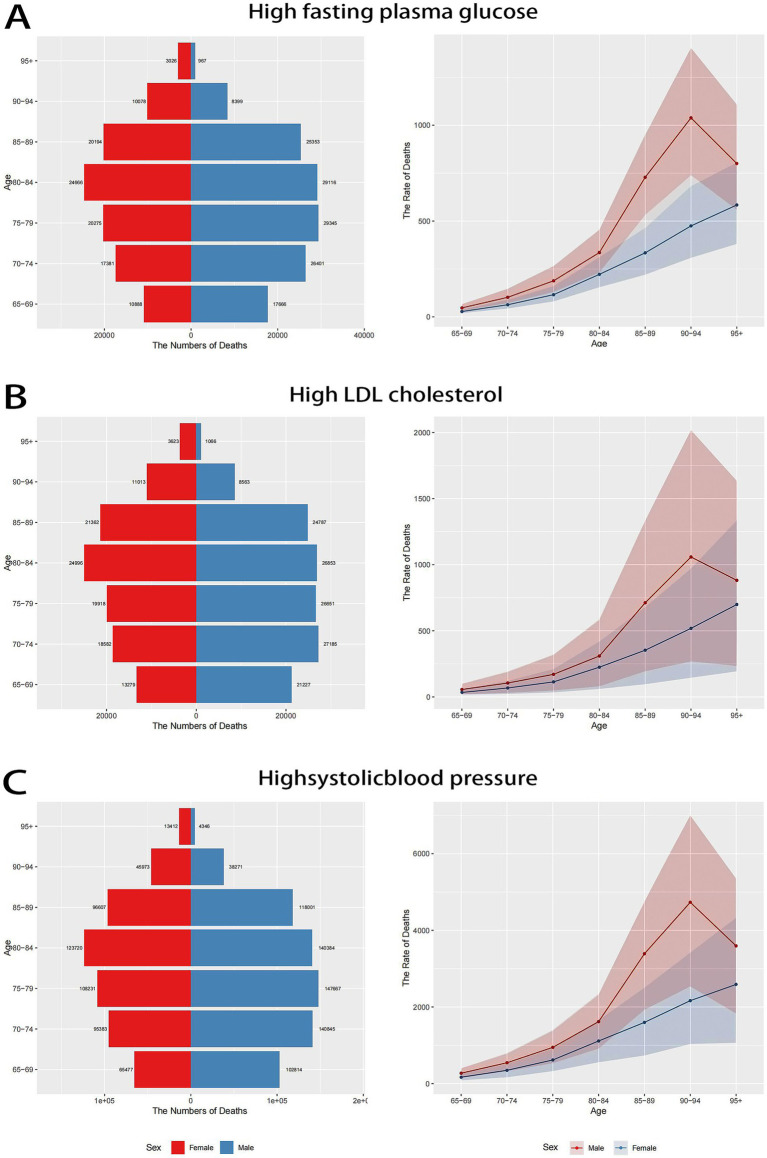
Comparison of deaths number and rate of stroke attribute to metabolic risks in males and females among people aged ≥65 years of different age groups in China in 2021. **(A)** Deaths number and rate of stroke attribute to high fasting plasma glucose; **(B)** deaths number and rate of stroke attribute to high LDL cholesterol; **(C)** deaths number and rate of stroke attribute to high systolic blood pressure.

In 2021, the burden of stroke exhibited a clear age-dependent trend caused by high fasting plasma glucose, high LDL cholesterol, and high systolic blood pressure (Figure 1). Across all metabolic risks, mortality and ASDR rose progressively with age, peaking in the oldest cohorts. The ASDR for stroke attributed to all three metabolic risk factors was highest in the 90–94 year age group. Men consistently demonstrated higher deaths number and ASDR than women across all age groups. Among the three risk factors, high systolic blood pressure contributed the most to stroke-related deaths and ASDR, followed by high fasting plasma glucose and high LDL cholesterol.

### Trends in stroke burden attributable to metabolic risks from 1992 to 2021

**Figure 2:**
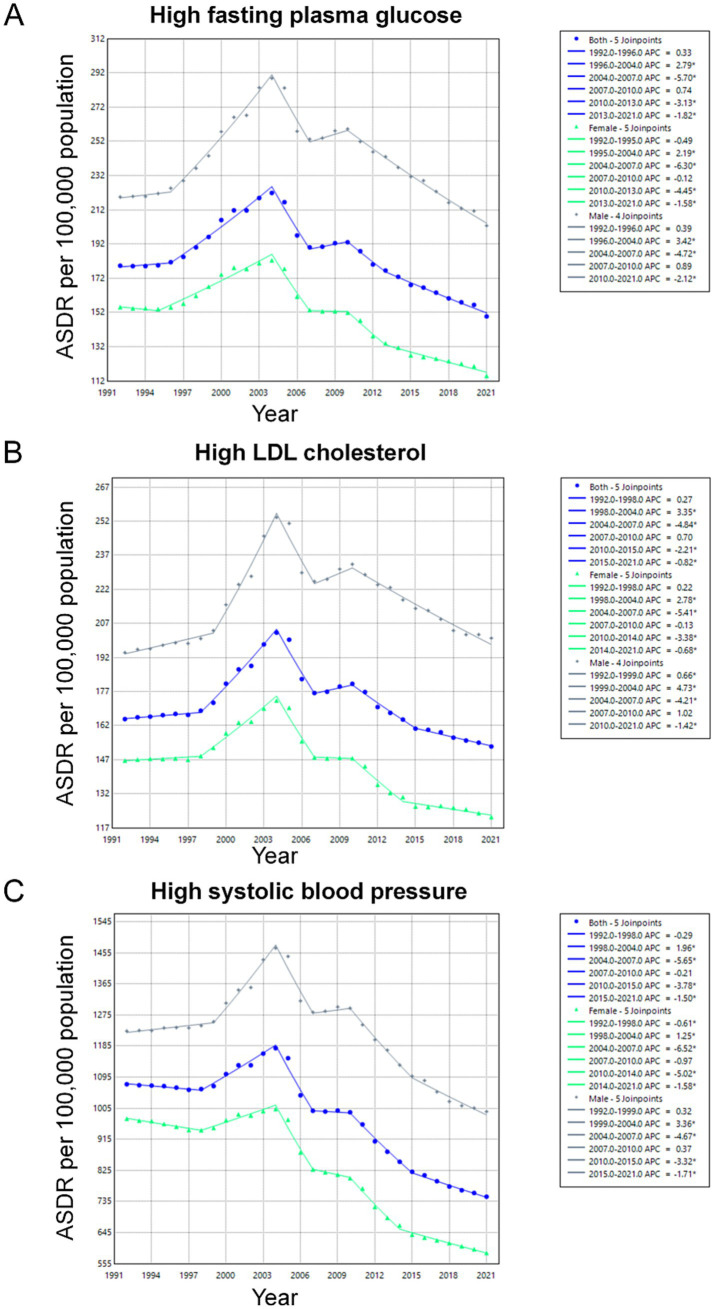
Trends in ASDR of Stroke Attributable to Metabolic Risks Among Individuals Aged 65 and Older in China, 1992–2021. The Joinpoint regression analysis illustrating the trends in ASDR for stroke due to key metabolic risk factors over the study period: **(A)** high fasting plasma glucose; **(B)** high LDL cholesterol; **(C)** high systolic blood pressure. ASDR, Age-Standardized Death Rates.

A steady decline in ASDR was observed for high fasting plasma glucose, high LDL cholesterol, and high systolic blood pressure (Figure 2). Analyses conducted over different time periods revealed that the ASDR for stroke due to high fasting plasma glucose remained stable from 1992 to 1996. Similarly, the ASDR for stroke due to high LDL cholesterol and high systolic blood pressure remained stable from 1992 to 1998. The ASDR for all three risk factors then showed a significant increase until 2004, when it peaked. From 2004 to 2007, the ASDR for all three risk factors decreased significantly. After a period of stability from 2007 to 2010, the ASDR for all three risk factors decreased significantly over the following 10 years. Further analyses stratified by gender subgroups revealed that the total number of deaths cases of stroke steadily increased in both genders, while the ASDR declined across all metabolic risks (Supplementary Figure 1). Similar to the 2021 age-stratified results, the number of deaths and ASDR attributed to the three risk factors were consistently higher in men than in women over the 30-year period from 1992 to 2021.

### Age-period-cohort effects on stroke mortality

**Figure 3:**
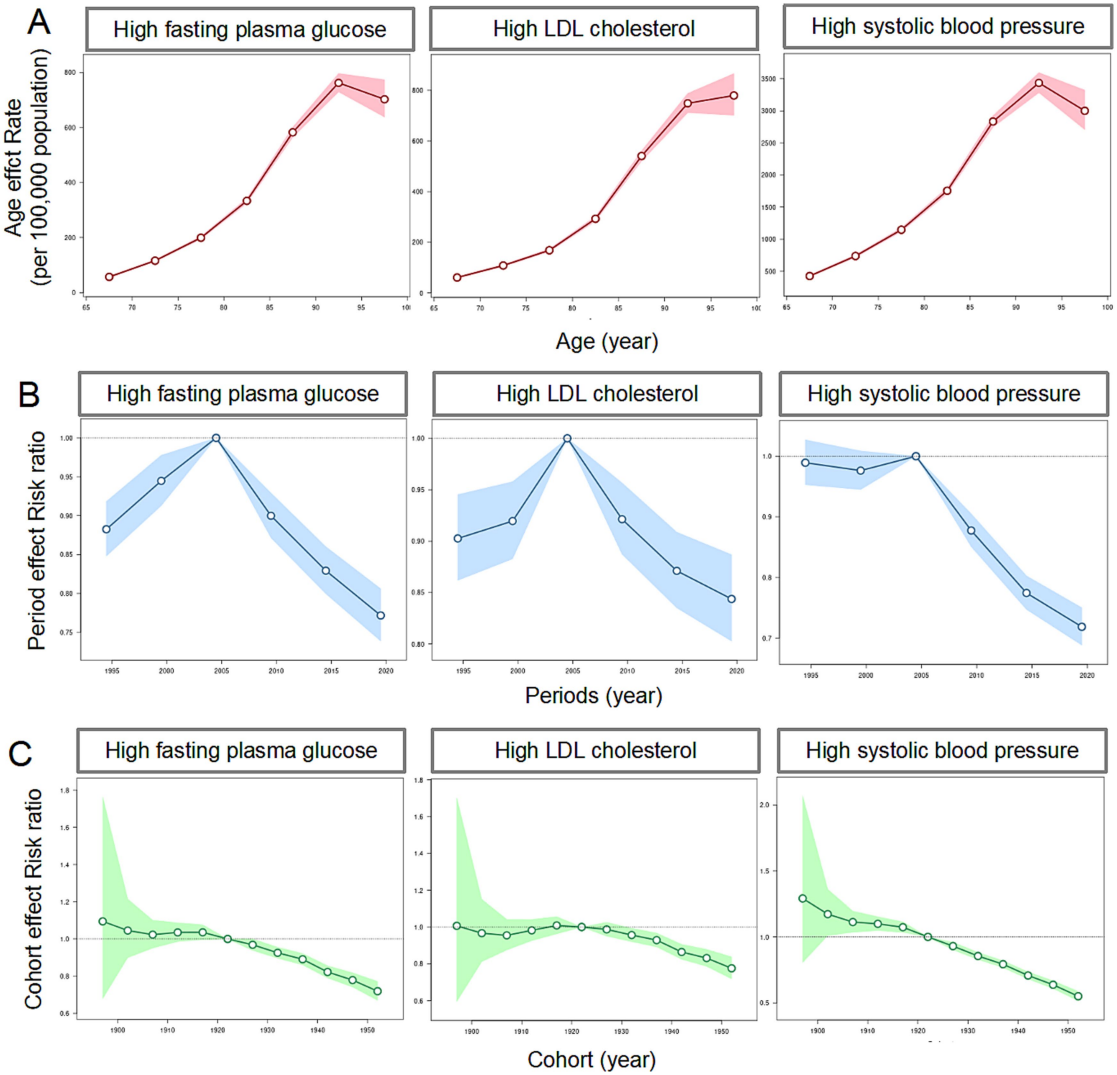
The age-period-cohort effects on the mortality of stroke attribute to metabolic risks among people aged ≥65 years in China. **(A)** Age effects on the mortality of stroke attribute to metabolic risks; **(B)** period effects on the mortality of stroke attribute to metabolic risks; **(C)** cohort effects on the mortality of stroke attribute to metabolic risks.

The age-period-cohort effects on stroke mortality associated with metabolic risks are illustrated in Figure 3, with all three metabolic risks showing similar patterns. The age-specific curves reveal a sharp increase in ASDR with advancing age, particularly for high SBP. Period effects indicate a gradual reduction in stroke mortality across successive time periods. The mortality risk in the HFPG and high LDL-C groups increased from 1992, peaked in 2005, and subsequently declined progressively. In contrast, the mortality risk in the high SBP group remained elevated since 1992, with a significant decrease observed from 2005 onwards. Cohort effects demonstrate higher mortality risks among individuals born in earlier decades.

### Predicted trends in stroke burden over the next decade

**Figure 4:**
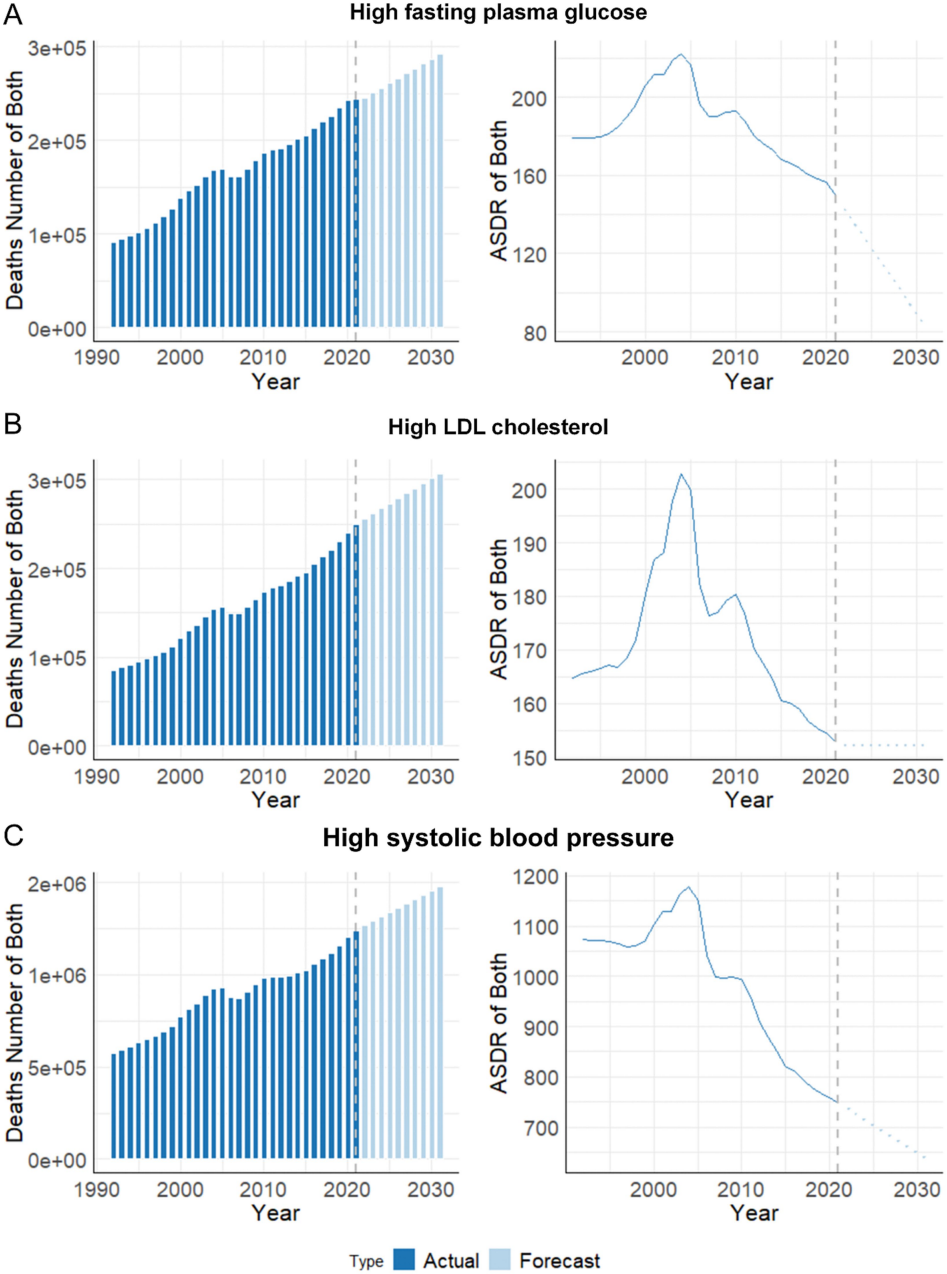
Predicted trends in deaths number and ASDR of stroke attribute to metabolic risks among people aged ≥ 65 years in China over the next 10 years (2022–2031). **(A)** High fasting plasma glucose; **(B)** High LDL cholesterol; **(C)** High systolic blood pressure. ASDR, age-standardized deaths rate.

The projections indicate that the absolute number of deaths associated with HFPG, high LDL-C, and high SBP is expected to continue rising (Figure 4). The optimized model was selected for modeling the number of deaths and the ASDR of stroke after being identified using the auto.arima() function (25). By 2031, the overall number of new stroke cases attributed to HFPG, high LDL-C, and high SBP among older adults is expected to increase to 291,662, 307,040, and 1,478,943, respectively. However, the ASDR attributed to these three metabolic risks is expected to decline until 2031. By 2031, the ASDR is projected to decrease to 82.14, 152.09, and 636.08, respectively.

## Discussion

This study offers an in-depth evaluation of the stroke burden attributable to metabolic risk factors, including HFPG, high LDL-C, and high SBP, among Chinese adults aged 65 years and older from 1992 to 2021, utilizing age-period-cohort analysis to elucidate their relative impacts and project deaths through 2031. Significantly, both the ASR of death and DALYs declined substantially over the past three decades, reflecting improvements in healthcare, prevention, and risk factor management, consistent with downward global trends enabled by targeted interventions like hypertension control, glycemic management, and lipid-lowering therapies (2, 4, 26). However, our findings highlight a critical dual challenge: despite this favorable decline in rates, the absolute number of deaths and DALYs continues to rise, driven primarily by population aging and the increasing prevalence of metabolic disorders, underscoring the persistent and growing burden in absolute terms (27).

Among the analyzed metabolic risks, high SBP has been identified as the predominant factor contributing to stroke-related deaths and DALYs, reinforcing existing literature that cites hypertension as the foremost modifiable risk factor for stroke globally (3, 27, 28). Even though there have been significant reductions in ASRs, the absolute burden of stroke remains substantial, emphasizing the urgent need for intensified blood pressure control, particularly in the elderly demographic. Similarly, HFPG and high LDL-C are also showing increased absolute burdens, propelled by the rising prevalence of diabetes and dyslipidemia in China, trends that are closely associated with rapid urbanization, lifestyle modifications, and an aging population (8, 29–31). The slower decline in HFPG and high LDL-C-attributable stroke mortality compared to high SBP may be attributed to differences in public health focus and treatment uptake. While hypertension management has been prioritized globally, leading to improved awareness, treatment, and control rates, hyperglycemia and dyslipidemia have received less attention (32–34).

Age-specific analyses have consistently shown an increase in stroke burden with advancing age, with a peak in individuals aged 75 years and older. This expected trend reflects the cumulative exposure to metabolic risks, co-existing comorbidities, and age-related physiological changes affecting vascular health (5, 6). In the United States, women aged 45–74 years have a substantially lower risk of stroke mortality than men—approximately 25–35% lower for Black women and 20% lower for White women. However, this advantage diminishes in older age groups, partly because elderly women tend to have a longer life expectancy, are more likely to live alone, and are at greater risk of social isolation (35). Similar to age-adjusted mortality rates, women also have a lower overall age-adjusted stroke incidence than men. A systematic review revealed that in Latin America and the Caribbean, the stroke incidence rate was higher in men than in women, with an incidence rate ratio of 1.12 (95% CI, 1.04–1.21) (36). In the Southeast Asia region, the age-standardized rates of stroke incidence and mortality were also lower in women compared to men (37). Furthermore, a cross-sectional study estimated that in 2020, 10.3 million males (95% CI, 10.1–10.5) and 7.5 million females (95% CI, 7.3–7.6), aged 40 years or older in China had experienced a stroke. Among them, 2.0 million males (95% CI, 1.9–2.1) and 1.4 million females (95% CI, 1.4–1.5) had a first-ever stroke (12). The observed higher burden in males compared to females across all risk factors aligns with existing data, which suggests higher rates of smoking, alcohol consumption, and uncontrolled hypertension among men (38, 39). These insights underscore the necessity for targeted prevention strategies that are tailored to specific sex-based risk profiles, especially among high-risk male groups.

The age-period-cohort analysis provides deeper insights into how demographic and temporal factors longitudinally affect stroke mortality. Age curves notably highlight the disproportionate vulnerability of older age groups, especially concerning high SBP. Period effects illustrate consistent reductions in stroke mortality over time, reflective of enhancements in healthcare systems, improved accessibility to treatments, and public health initiatives aimed at metabolic risk reduction (40). However, cohort effects underscore the enduring impact of historical exposures to adverse metabolic conditions, particularly among older generations who previously had limited access to advanced healthcare and risk management (41).

Looking ahead, future projections suggest an ongoing increase in the absolute number of stroke deaths related to metabolic risks through the next decade (2022–2031), primarily driven by demographic aging. Although reductions in ASDR are anticipated, the expanding elderly population is expected to further compound the stroke burden, necessitating continued efforts in risk factor management and healthcare system enhancements (42). Gender-specific and age-focused interventions will be pivotal in maximizing the effectiveness of these strategies, ensuring that blood pressure, glucose levels, and lipid management remain central priorities, alongside broader lifestyle interventions that address obesity, diet, and physical inactivity (30, 43).

The strengths of this study include its extensive geographic coverage and extended duration, supported by a robust dataset. By estimating ASDR for stroke attributable to metabolic risks among individuals aged 65 years and older, we effectively address age structure heterogeneity. Our use of age-time-cohort modeling further distinguishes the independent effects of age, period, and cohort on stroke mortality risk. Nevertheless, this study has several limitations. First, as the analysis is based on estimates from the GBD 2021 study, it is subject to the inherent limitations of large-scale modeling approaches. These include reliance on statistical assumptions, prior distributions, and potential parameter uncertainties, particularly in regions or time periods with sparse or low-quality primary data. Second, the quality and availability of input data vary across countries and years, which may result in inconsistencies in the accuracy of estimates. For instance, the lack of local epidemiological studies, possible misclassification or underdiagnosis of stroke, and insufficient data on certain risk factors may affect the robustness of the findings in some settings. Third, although the study focused on three major metabolic risk factors, it did not explicitly account for important behavioral risks—such as smoking, alcohol use, physical inactivity, and dietary habits—which are known to interact with metabolic factors in influencing stroke outcomes. Fourth, potential confounding due to comorbid conditions was not adjusted for in the aggregate-level data. Finally, we did not analyze subnational variations within China, which may limit the generalizability of our findings to regional or vulnerable subpopulations.

## Conclusion

In conclusion, while notable progress has been made in reducing ASRs of stroke attributable to metabolic risks, the absolute burden continues to escalate due to demographic shifts and the rising prevalence of metabolic disorders. Future public health initiatives must prioritize the management of high SBP, HFPG, and high LDL-C, incorporating tailored strategies that address age- and sex-specific disparities. Strengthening healthcare systems to accommodate an aging population and effectively mitigating the impact of metabolic risks will be crucial for further reducing the stroke burden in China.

## Data Availability

Publicly available datasets were analyzed in this study. The data used in this study are available from the Global Burden of Diseases, Injuries, and Risk Factors Study (GBD) 2021 (https://ghdx.healthdata.org/gbd-2021).

## Footnotes

^1^ http://ghdx.healthdata.org/

## Supplementary material

Supplementary Figure 1 Trends of deaths number and ASDR of stroke attribute to metabolic risks among people aged ≥65 years in China, as well as for males and females from 1991 to 2021. **(A)** High fasting plasma glucose; **(B)** high LDL cholesterol; (C) High systolic blood pressure. ASDR, age-standardized deaths rate.
