# Supplementary figures and images for: Temporal trends and epidemiological impact of metabolic risk factors on stroke burden in Chinese individuals aged 65 and older, 1992–2021
Source: Front Neurol. 2025 Jul 29;16:1607823. doi: 10.3389/fneur.2025.1607823 (PMC12341581; doi:10.3389/fneur.2025.1607823)

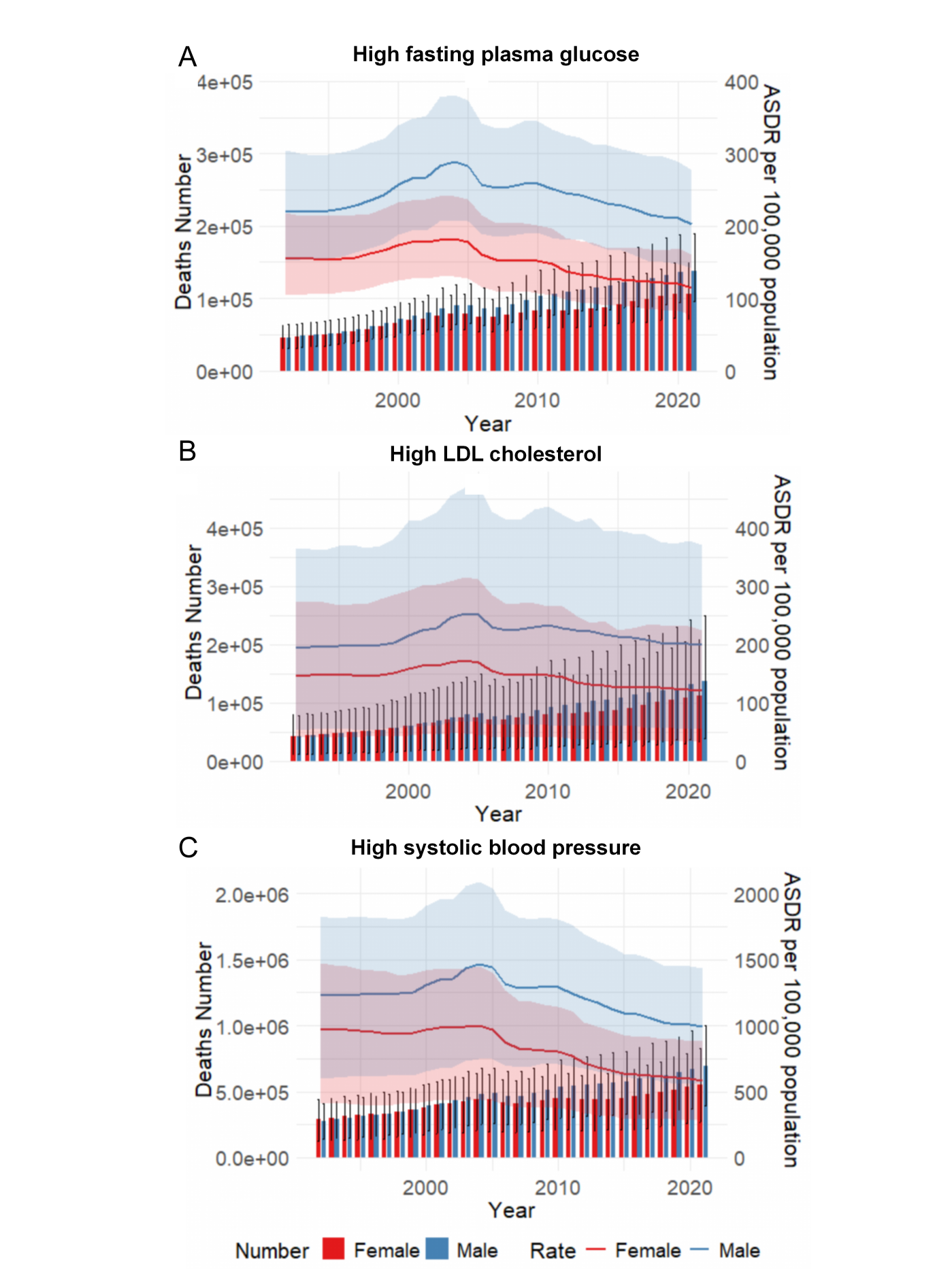

Supplement: Supplementary Figure 1 — Trends of deaths number and ASDR of stroke attribute to metabolic risks among people aged ≥65 years in China, as well as for males and females from 1991 to 2021. (A) High fasting plasma glucose; (B) high LDL cholesterol; (C) High systolic blood pressure. ASDR, age-standardized deaths rate. [file Image_1.tif]
